# Whole-body vibration training versus conventional balance training in patients with severe COPD—a randomized, controlled trial

**DOI:** 10.1186/s12931-021-01688-x

**Published:** 2021-05-04

**Authors:** Rainer Gloeckl, Tessa Schneeberger, Daniela Leitl, Tobias Reinold, Christoph Nell, Inga Jarosch, Klaus Kenn, Andreas R. Koczulla

**Affiliations:** 1 Department of Pulmonary Rehabilitation, Philipps-University of Marburg, German Center for Lung Research, Malterhoeh 1, 83471 Schoenau Am Koenigssee, Marburg, Germany; 2 Institute for Pulmonary Rehabilitation Research, Schoen Klinik Berchtesgadener Land – Schoenau am Koenigssee, Königsee, Germany; 3 Department of Prevention, Rehabilitation and Sports Medicine, Technical University of Munich, Munich, Germany; 4 Department of Internal Medicine, Division of Pulmonary Diseases, Philipps University of Marburg, Marburg, Germany; 5 Teaching Hospital, Paracelsus Medical University, Salzburg, Austria

**Keywords:** Chronic obstructive pulmonary disease, Pulmonary rehabilitation, Exercise, Neuromuscular power, Force measurement platform, Vibration platform

## Abstract

**Background:**

Whole-body vibration training (WBV) performed on a vibration platform can significantly improve physical performance in patients with chronic obstructive pulmonary disease. It has been suggested that an important mechanism of this improvement is based on an improvement in balance. Therefore, the aim of this study was to investigate the effects of WBV compared to conventional balance training.

**Methods:**

48 patients with severe COPD (FEV_1_: 37 ± 7%predicted) and low exercise performance (6 min walk distance (6MWD): 55 ± 10%predicted) were included in this randomized controlled trial during a 3 week inpatient pulmonary rehabilitation. All patients completed a standardized endurance and strength training program. Additionally, patients performed 4 different balance exercises 3x/week for 2 sets of 1 min each, either on a vibration platform (Galileo) at varying frequencies (5–26 Hz) (WBV) or on a conventional balance board (BAL). The primary outcome parameter was the change in balance performance during a semi tandem stance with closed eyes assessed on a force measurement platform. Muscular power during a countermovement jump, the 6MWD, and 4 m gait speed test (4MGST) were secondary outcomes. Non-parametric tests were used for statistical analyses.

**Results:**

Static balance performance improved significantly more (*p* = 0.032) in favor of WBV (path length during semi-tandem stand: − 168 ± 231 mm vs. + 1 ± 234 mm). Muscular power also increased significantly more (*p* = 0.001) in the WBV group (+ 2.3 ± 2.5 W/kg vs. − 0.1 ± 2.0 W/kg). 6MWD improved to a similar extent in both groups (WBV: 48 ± 46 m, *p* < 0.001 vs. BAL: 38 ± 32 m; *p* < 0.001) whereas the 4MGST increased significantly only in the WBV-group (0.08 ± 0.14 m/s^2^, *p* = 0.018 vs. 0.01 ± 0.11 m/s^2^, *p* = 0.71).

**Conclusions:**

WBV can improve balance performance and muscular power significantly more compared to conventional balance training.

*Trial registration:* Clinical-Trials registration number: NCT03157986; date of registration: May 17, 2017. https://clinicaltrials.gov/ct2/results?cond=&term=NCT03157986&cntry=&state=&city=&dist =

**Supplementary Information:**

The online version contains supplementary material available at 10.1186/s12931-021-01688-x.

## Introduction

Whole-body vibration training (WBV) is a training modality where a subject stands on a vibration platform that induces sinusoidal oscillations to the body which evolve reflex-induced muscle contractions [1]. It has been shown that neuromuscular activity during WBV is increased compared to similar exercises without vibration [2, 3]. Therefore, within the past two decades, there has been increasing interest in the use of WBV as a training intervention in several therapeutically areas like chronic low back pain [4], osteoporosis [5], neurological disorders [6], or geriatric rehabilitation [7]. There is also increasing evidence that WBV is a beneficial exercise modality in patients with chronic obstructive pulmonary disease [8]. In a recent study from our workgroup, we found that improvements in exercise performance following a WBV training program were related to improvements in balance performance and muscular power output [9]. Furthermore, these neuromuscular adaptations seemed to be an important mechanism of improving exercise performance especially in COPD patients with impaired balance performance and low exercise performance [9]. Currently, there is a large body of evidence showing that postural control and balance performance are impaired in COPD compared to healthy age-matched controls [10, 11]. This leads to an increased risk for falls in COPD [12]. A large cohort study analyzing more than 200.000 subjects has shown that COPD patients are 55% more likely to have fall incidents compared to non-COPD subjects [13]. Since falls are associated with an increased risk of injuries, injury-related disability, and even an increased risk of all-cause mortality, improving balance performance and preventing falls has become an important treatment target in COPD [14]. Thus, measures of balance performance are also recommended by the current ATS/ERS pulmonary rehabilitation guidelines to be included in the clinical assessment of patients with COPD [15].

Hence, the aim of our study was to investigate the effects of a balance training using WBV vs. a conventional balance training on balance performance and muscular power in COPD patients with an impaired physical status.

## Methods

### Study design

Patients admitted to an inpatient rehabilitation program at the Schoen Klinik Berchtesgadener Land (Schoenau am Koenigssee, Germany) were screened for eligibility to participate in this randomized controlled trial. Patients were recruited between May 2017 and August 2019. This study was submitted to the Clinical Trials Registry (www.clinicaltrials.gov, NCT03157986) and approved by the Ethics Committee of the Philipps-University of Marburg (approval number: 27/17).

### Study population

Inclusion criteria were: age between 50 and 80 years, confirmed diagnosis of COPD stage III or IV according to the Global Initiative for Chronic Obstructive Lung Disease (GOLD) [16] guidelines, a 6 min walk distance (6MWD) < 70% predicted, and providing a written informed consent [17]. Patients with a current acute COPD exacerbation, a carbon dioxide pressure ≥ 45 mmHg at rest, or any contraindications for WBV (e.g. an artificial joint in the lower extremities) were excluded.

### Intervention

Patients participated in a 3 week comprehensive multimodal and multidisciplinary inpatient pulmonary rehabilitation (PR). The PR program was provided on 6 days per week consisting of medical care, endurance training, strength training, respiratory physiotherapy, education, nutritional and psychological counseling. For detailed information on the standardized endurance and strength training program see Additional file 1: Tables S1).

For this study, all patients performed a supplemental supervised balance training on 3 non-consecutive days per week (Mon/Wed/Fri). Patients were randomized and allocated to either a WBV group or a conventional balance training group (BAL). The WBV group performed a balance training on a side-alternating vibration platform (Galileo, Novotec, Medical GmbH, Pforzheim Germany) at varying frequencies (5–26 Hz) and 4–5 mm peak-to-peak displacement (see Table 1 and Additional file 1: Table S2 for a detailed description of WBV settings). The varying frequencies were used to provide different stimuli to the patients´ motor control. Patients in the BAL group performed the same exercises on a conventional balance board.

**Table 1:** Balance exercises and vibration plate settings

	Exercises (2 × 1 min each)	WBV group frequencies	Conventional balance training	Variations
1	Dynamic squat exercise	Mon: 26 Hz Wed: 18 Hz Fri: 22 Hz	Balance board	Slight finger contact on a handlebar
2	Dynamic heel raises	Mon: 22 Hz Wed: 26 Hz Fri: 18 Hz	Balance board	Free standing
3	Static one-leg stance	Mon: 15 Hz Wed: 10 Hz Fri: 5 Hz	Balance board	Additional arm/leg movements
4	Dynamic lunge step	Mon: 18 Hz Wed: 22 Hz Fri: 26 Hz	Balance board	Throwing balls

*Mon* Monday, *Wed* Wednesday, *Fri* Friday

**Fig. 1:**
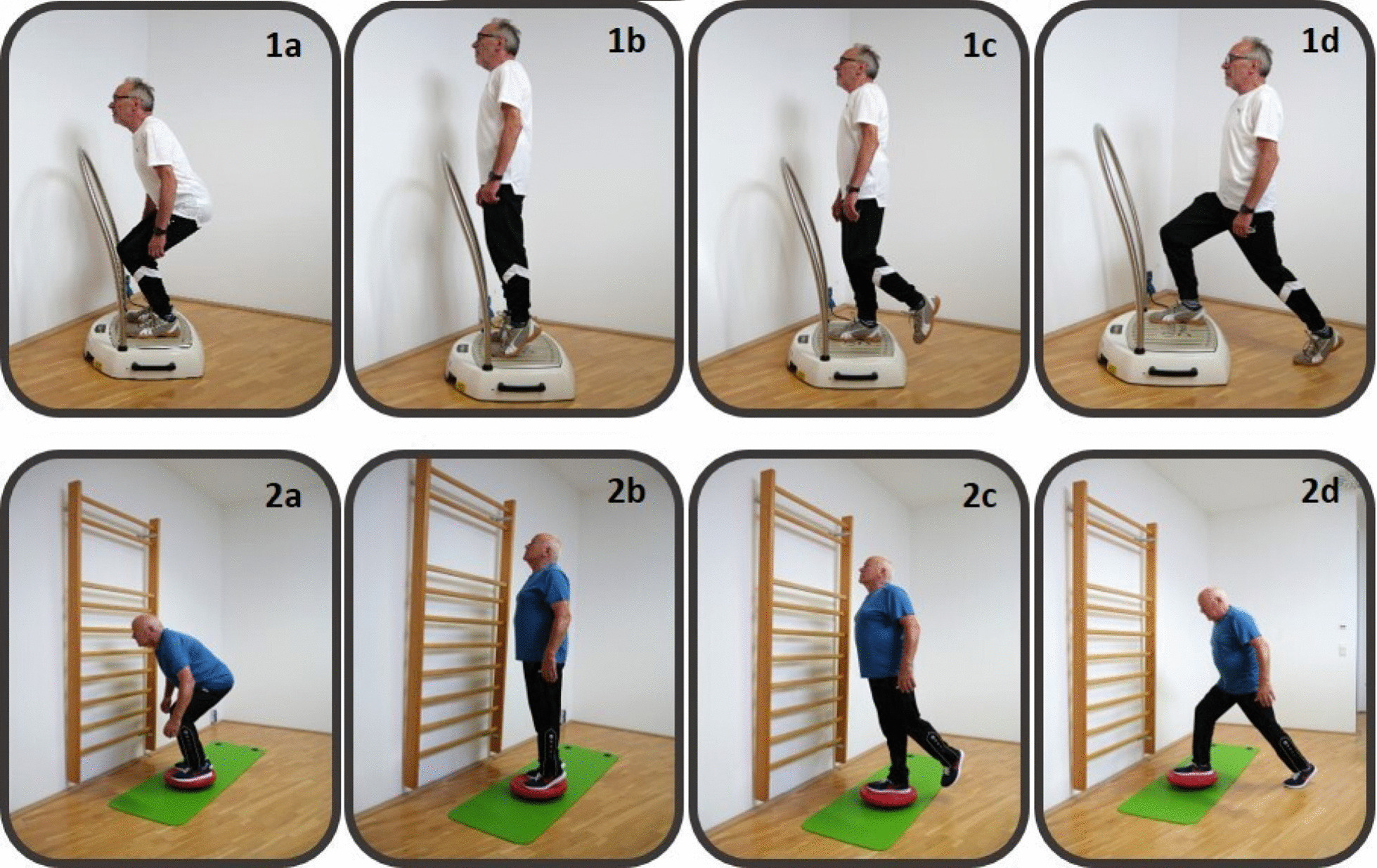
Exercises performed during whole-body vibration balance training. Whole body vibration training and conventional balance training performed for 2 sets of 1 min per exercise and training session: **a** dynamic squats, **b** dynamic heel raises, **c** static one-leg stance, and **d** dynamic lunge step. (patients provided written informed consent for the use of these figures)

Balance training sessions were similar between groups except for the surface and consisted of four exercises as described in Table 1 & Fig. 1. Each balance training session took about 20 min (including short breaks of 1-min duration between each exercise). Patients were instructed to perform exercises with slow-motion movements (3 s concentric and eccentric). When a participant was able to perform an exercise with only little instability, the difficulty was progressively increased by adding more challenging conditions (e.g. from slight finger contact on a handlebar and freestanding to closed eyes and additional arm movements to irritate balance ability). The aim was to reach an individual level of difficulty that forced patients for continuous counter-movements. In the WBV group exercise intensity varied also by using different vibration frequencies between 5 to 26 Hz during each session (see Table 1). All balance training sessions in both groups were individually supervised by an experienced therapist that corrected and adapted exercise difficulty to the patients individual performance.

### Outcomes and measures

#### Primary outcome—balance performance

**Fig. 2:**
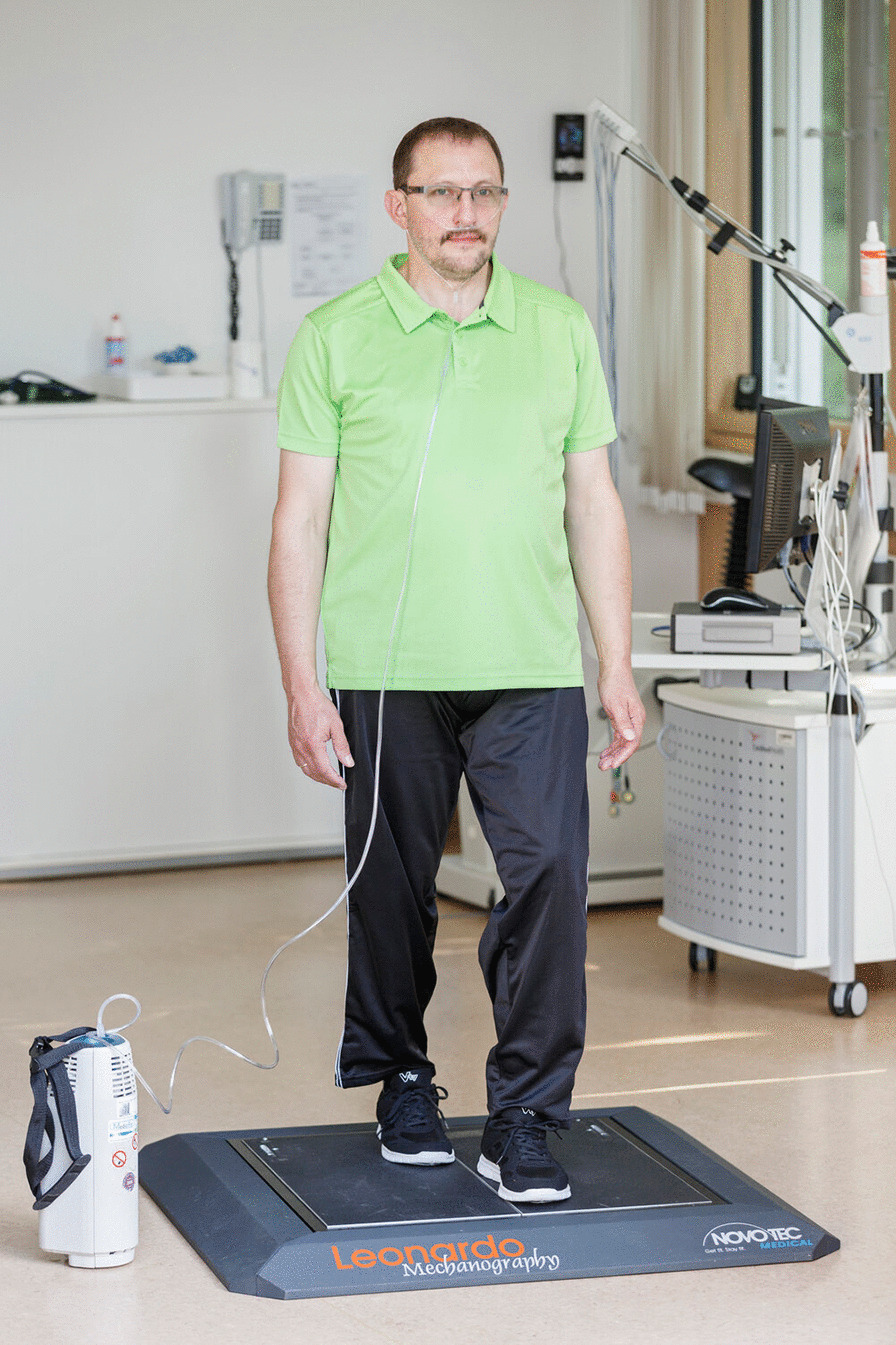
COPD Patient performing a semi-tandem stance balance test on a force measurement platform. Patient provided written informed consent for the use of this picture

The primary outcome parameter of the study was the change in balance performance during semi tandem stance (Fig. 2) with closed eyes. Further standing positions to test the postural balance were Romberg stance (eyes closed) and one-leg stance (eyes open). For all 3 balance tests patients were instructed to stand as still as possible for 10 s. The best out of three attempts was used for analysis. The balance tests were assessed using a ground reaction force platform (Leonardo Mechanograph, Novotec Medical, Pforzheim, Germany) with 8 integrated force sensors (800 Hz each) to calculate the center of force [18]. The outcome “absolute path length” in mm represents a better stability with lower values.

#### Secondary outcomes

##### Neuromuscular performance

To measure muscular power a countermovement jump was performed on the Leonardo platform [19]. Patients were asked to jump as high as possible with using arm-swing [20]. The best test out of three jumps was used for analysis. Peak Watt per kg body weight was used as outcome.

##### 6-min walk distance

The 6-min walk test (6-MWT) was performed following the ATS/ERS guidelines [21] with the best out of two tests being used for analysis. The minimal important difference in COPD is estimated to be 30 m [21].

##### 4-m gait speed test

A 4-m gait speed test has been performed according to Kon et al. [22]. The minimal important difference in COPD is estimated at 0.11 m/s [23].

##### Muscular strength

A dynamometer (MicroFET2, Hoggan, Scientific LLC, UT) was fixed in a leg curl device to measure peak isometric knee extension strength at 90° knee angle.

##### Sit to stand tests (STST)

A five-repetition STST (outcome: test duration in seconds) [24] and a 1 min STST (outcome: number of repetitions) [25] were performed from a 46 cm height bench with arms crossed in front of the chest.

### Sample size calculation

The a priori sample size computation based on the results of a former trial [9] and included the following assumptions: changes in the APL of the semi-tandem stance with closed eyes (= primary outcome) of -272 ± 369 mm (WBV) and 76 ± 277 mm (control), power 95%, alpha of 5% and two-sided, independent t-test. Based on these assumptions, a sample size of 24 per group was necessary to achieve this power at this effect size.

### Randomization and allocation concealment

Stratification for randomization was done according to balance performance using a threshold of 750 mm absolute path length during the baseline semi-tandem stance. The investigator responsible for patient recruitment received group allocation by a third party picking a sealed envelope which contained group allocation.

### Blinding

Blinding of the study participants was not possible within the study setting due to the nature of the intervention. However, the outcome assessors and the statistician were blinded to the group allocation.

### Statistical methods

Results were provided by mean values ± SD or 95%CI. For comparing pre to post PR effects, a two-tailed Wilcoxon rank-sum test was applied. The Mann–Whitney U-test was used to compare the between-group differences. The significance level was set at *p* < 0.05. Regression models with a forward variable selection algorithm were used to test for significant predictors of change following the intervention. Statistical analyses were performed using SPSS 23 (IBM, USA).

## Results

**Fig. 3:**
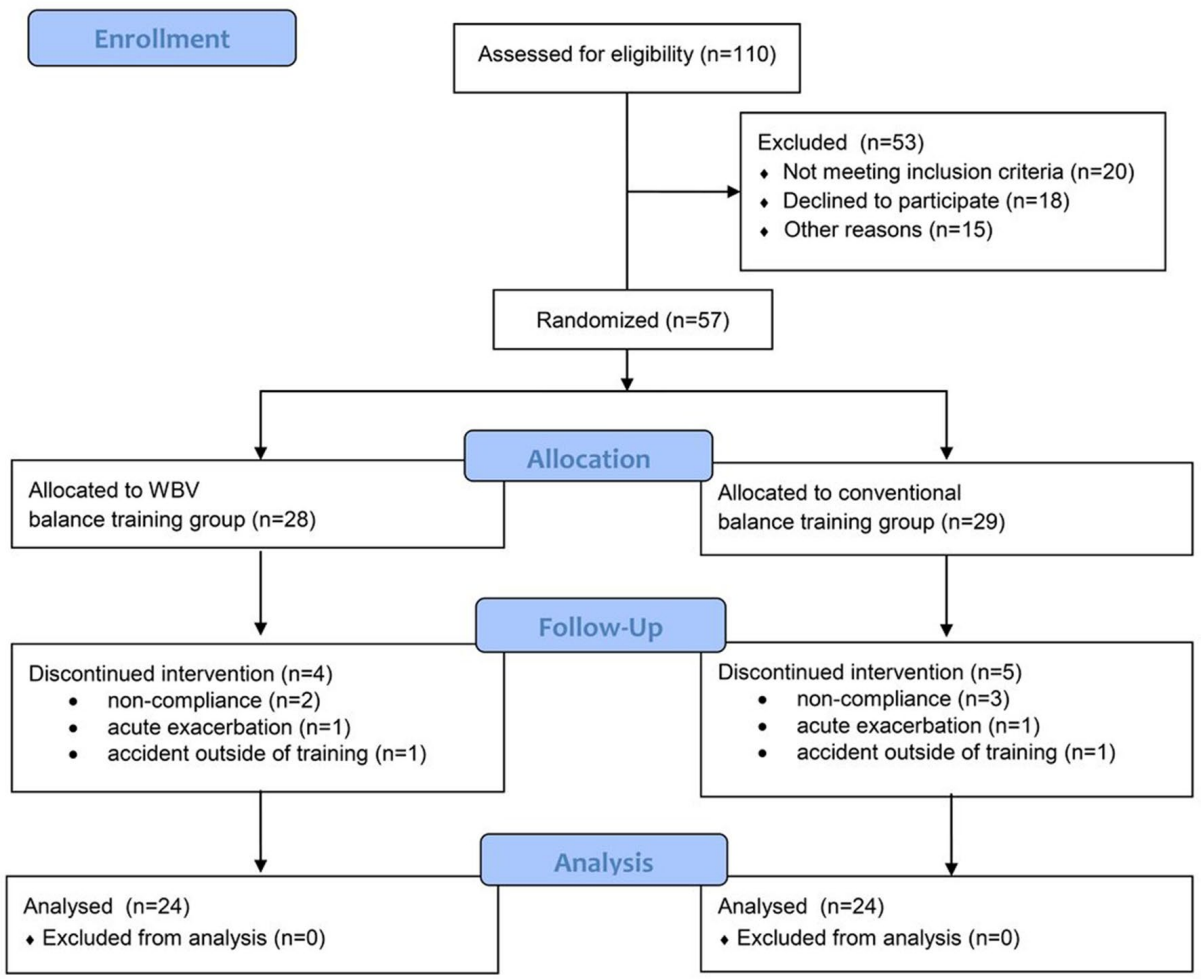
Consort flow diagram

**Fig. 4:**
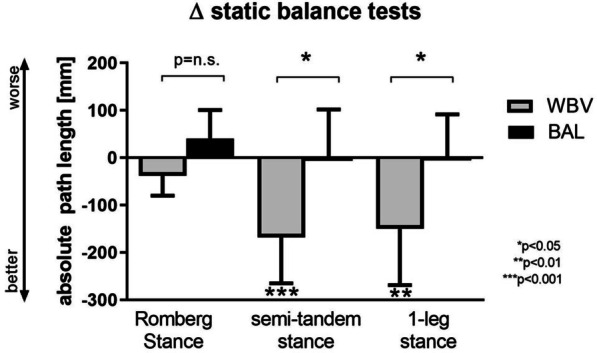
Changes in balance performance during Romberg stance, semi-tandem stance, and 1-leg stance following a whole-body vibration (WBV) balance training or conventional balance training (BAL)

Fifty-seven out of 110 eligible patients met the inclusion criteria and were randomized to the trial. Nine patients dropped out of the study (for reasons see flow chart in Fig. 3) and 48 patients completed all assessments. Patients had severe airflow obstruction (FEV1: 37 ± 7%predicted) and impaired exercise capacity (6-MWD: 354 ± 70 m, 55 ± 10%predicted). For more baseline measures see Table 2. Patients performed on average 8 ± 1 out of a maximum of nine possible balance training sessions in the WBV group and 7 ± 1 sessions in the control group. The primary outcome (change in APL during semi-tandem stance) improved significantly more in favor of the WBV group (between-group difference: 167 mm, p = 0.032) with a medium effect size (cohen´s d 0.72; Fig. 4). Patients in the control group did not significantly improve in any balance test. Another measure of neuromuscular performance, the countermovement jump also improved significantly more in favor of the WBV group (+ 2.3 W/kg vs. − 0.1 W/kg, *p* = 0.001; Table 3). The 4 m gait speed test improved significantly only in the WBV group (0.08 m/s^2^, *p* = 0.018 vs. 0.01 m/s^2^, *p* = 0.715). However, walking performance during the 6-MWT increased similarly in both groups (see Additional file 1: Figures S1–S4). It was not possible to set up a regression model, since no stable model could not be achieved, which might be related to the small number of patients.

**Table 2:** Baseline characteristics

	WBV balance training group	conventional balance training group	*p*-values
General			
n	24	24	–
Age, ys	65 ± 7	66 ± 8	0.415
BMI, kg/m^2^	25.3 ± 5.1	26.0 ± 5.2	0.398
LTOT, n [%]	11 (46%)	8 (33%)	0.376
FEV_1_, %predicted^#^	36.2 ± 8.4	38.0 ± 6.3	0.466
RV, %predicted^#^	218.6 ± 40.4	233.4 ± 37.1	0.301
pO_2_ at rest and ambient air, mmHg	61.5 ± 5.5	63.8 ± 4.1	0.155
Comorbidities, n	2.5 [0–5]	2.4 [0–6]	0.756
Falls during the previous year, n (%)	2 (8%)	5 (21%)	0.389
Static balance tests			
Romberg stance/eyes closed, APL [mm]	352 ± 197	387 ± 225	0.578
Semi tandem stance/eyes closed, APL [mm]	800 ± 268	887 ± 417	0.750
One-leg stance/eyes open, APL [mm]	786 ± 356	731 ± 276	0.844
Exercise performance tests			
6MWD, m	349 ± 68	360 ± 73	0.621
6MWD, %predicted	53 ± 10	57 ± 10	0.155
Knee extension, peak force [N]	267 ± 74	282 ± 99	0.733
Knee extension, peak force [%predicted]	79 ± 17	87 ± 19	0.249
Countermovement jump [W/kg]	23.9 ± 5.1	23.3 ± 6.0	0.517
5-repetition sit-to-stand test [sec.]	13.4 ± 3.9	12.3 ± 4.8	0.091
1 min sit-to-stand test [rep]	18.3 ± 4.9	19.8 ± 6.5	0.109
4 m gait speed test [m/s^2^]	0.90 ± 0.15	0.97 ± 0.15	0.152

Data are presented as mean ± SD or [min/max]

*6MWD* 6-min walk distance, *APL* absolute path length, *BMI* Body-Mass-Index, *FEV1* forced expiratory volume in 1 s, *LTOT* long-term oxygen therapy, *pO2* partial oxygen pressure, *WBV* whole-body vibration training

^#^lung function was measured by bodyplethysmography (Master Screen Body, Jaeger, Germany) using reference equations from the Global Lung Function Initiative [26]

**Table 3:** Changes in outcome measures following 3 weeks of PR with whole body vibration (WBV) balance training or conventional balance training

	WBV balance training (n=24)	p	conventional balance training (n=24)	*p*	Between group difference	*p*	Effect size Cohen´s d
Balance tests							
Semi tandem stance/eyes closed, APL [mm]	− 168 (− 265–71)	<0.001	1 (− 100–102)	0.627	167 (32–305)	0.032	0.72
Romberg stance/eyes closed, APL [mm]	− 38 (− 80–4)	0.103	41 (− 19–101)	0.241	78 (7–150)	0.073	0.02
One-leg stance/eyes open, APL [mm]	− 150 (− 269–− 31)	0.005	2 (− 88–91)	0.744	152 (4–300)	0.017	0.68
Exercise performance tests							
6MWD, m	47.9 (28.2–67.5)	<0.001	38.4 (24.6–52.2)	<0.001	− 9.5 (− 33.0–14.0)	0.250	0.23
Knee extension, peak force [N]	35.1 (16.6–53.6)	0.001	25.9 (15.3–36.4)	<0.001	− 9.2 (− 29.4–10.9)	0.406	0.28
Countermovement jump [W/kg]	2.3 (1.1–3.4)	0.001	− 0.1 (− 0.9–0.8)	0.879	− 2.3 (− 3.7–− 1.0)	0.001	1.02
5-repetition sit-to-stand test [sec.]	− 2.5 (− 3.7–− 1.2)	0.001	− 1.6 (− 3.0–− 0.2)	0.012	0.9 (− 0.9–2.8)	0.072	0.30
1 min sit-to-stand test [rep]	3.6 (2.3–4.9)	<0.001	2.7 (1.2–4.3)	0.004	− 0.9 (− 2.9–1.1)	0.740	0.26
4 m gait speed test [m/s²]	0.08 (0.02–0.14)	0.018	0.01 (− 0.04–0.06)	0.715	− 0.07 (− 0.15–0.01)	0.107	0.56

Values are presented as mean and 95% CI

*6MWD* 6 minute walk distance, *APL* absolute path length, *BMI* Body-Mass-Index

No adverse event related to the training protocol was observed.

## Discussion

In our study, we found that WBV improved balance and neuromuscular performance significantly more compared to conventional balance training in COPD patients. It is known from former studies that in COPD patients postural control is impaired and gait parameters are altered compared to healthy age-matched controls [10, 11, 27]. Furthermore, COPD patients perceive an increased fear of falls than non-COPD individuals [27]. These impairments are of clinically relevance because they are associated with a lower functional performance and independence in activities of daily living [10]. COPD patients in our study had a 50% reduced balance performance compared to healthy elderly subjects [28]. This magnitude of balance impairment was similar to the one reported earlier in COPD [9]. A recent meta-analysis has identified several independent risk factors (like age, falls history, balance impairment, supplemental oxygen etc.) for falls in stable COPD [29]. However, impaired balance performance was the only risk factor that has the potential to improve.

Two recent systematic reviews concluded that general exercise training interventions during PR can improve balance performance in COPD [30, 31]. Furthermore, PR including a specific balance training program seemed to have the largest effect on balance [31]. A randomized, controlled trial by Beauchamp et al. [32] compared a conventional PR program (including general exercise training) with PR plus balance training (3x/week à 30 min for 6 weeks). The authors concluded that the addition of a specific balance training program significantly improved balance performance and self-reported physical function in patients with moderate to severe COPD. In contrast, patients in the conventional balance training group in our study did not improve balance performance (only patients in the WBV group did). This difference might be related to the different balance assessment methods (clinical balance tests like Berg Balance Scale vs. objective measures by a force measurement platform in the current study). Furthermore, the longer exercise duration (30 vs. 20 min per session) and intervention period (6 vs. 3 weeks) might have contributed to this difference. There is some evidence that greater benefits in balance performance can be achieved by higher doses of exercise [33]. Also, our conventional balance training program was strictly limited to the same four exercises on the balance board and was not extended to other exercises or further unstable surfaces. However, this was chosen to keep the exercise content in the two groups as standardized as possible. Furthermore, the combination of a conventional balance training program in addition to a general exercise training might have alleviated the balance outcomes since general exercise training itself has a very strong training effect [34]. However, interestingly balance training performed on a WBV platform was able to improve balance performance significantly even after such a short training period.

Former studies in older adults have already shown that WBV improves objectively measured balance performance [35] as well as self-perceived balance confidence [36]. Furthermore, a randomized, controlled trial by Stolzenberg et al. used a similar methodology like in the current study (strength training plus conventional balance training or WBV) in 55 postmenopausal women with low bone density [28]. It was concluded that combining strength training with WBV improved neuromuscular performance significantly more than strength training plus conventional balance training. Also, a recent systematic review and meta-analysis (10 studies including 557 subjects) summarized that WBV significantly improved functional mobility in elderly subjects [37]. It was hypothesized that these improvements could be useful for the tasks of daily living [37].

Currently, the underlying mechanisms for the WBV benefits on neuromuscular function are not fully understood yet [38, 39]. One of the most established explanations is that muscle contractions during WBV are induced by passive stretch reflexes [40, 41]. The micro-movements during WBV facilitate the excitability of the spinal reflex [42] compared to voluntary muscle control during conventional exercise training. Marin and colleagues have shown that the vastus lateralis electromyographical activity increased by 57% when subjects stood in a squatting position on a WBV platform compared to an isometric squatting position without WBV [43].

Beyond these significant benefits of WBV on neuromuscular performance, we did not find a significant difference in 6-MWD. This might be related to the reason, that the 6-MWT is not a highly sensitive test to detect changes especially between two active training interventions that are very similar [34, 44]. Interestingly, the 4-m gait speed test improved significantly only in the WBV group. This test is more suitable to reflect a patient's usual walking speed. Furthermore, the 4-m gait speed test is known as a surrogate marker of physical frailty [45]. Peak quadriceps force improved similarly in both groups what is in line with findings from former studies [9, 46]. Since WBV is not inducing a heavy muscular load during exercise a difference in muscular force was not expected.

Our study has some limitations that need to be discussed. First, we only included COPD patients with impaired exercise performance (6MWD < 70% predicted) what might limit the generalizability of our findings. However, we have chosen to do so because it is known that WBV has no additional effect on neuromuscular performance in well-trained athletes [47] and has only little effect in COPD patients with preserved exercise performance [9]. Therefore, WBV seems especially beneficial in subjects with impaired exercise and balance performance. The long-term maintenance of WBV training and its benefits in COPD e.g. on the risk of falls were not investigated and remain unknown. However, there is evidence that regular WBV over 8 to 12 months significantly reduced the risk of falls by 33% in subjects older than 50 years [48, 49]

A strength of our study is that balance and neuromuscular performance were objectively measured by a well-validated force measurement platform and standardized test procedures.

## Conclusions

To summarize, studies on the effects of exercise interventions on balance in COPD are still scarce, and more high-quality research is required [30]. In our study, we found that balance training performed on a WBV platform is superior to improve objectively measured balance performance and muscular power compared to conventional balance board training in patients with severe COPD and functional impairments.

## Supplementary Information

**Additional file 1.** Additional tables and figures.

## Data Availability

The datasets generated and/or analyzed during the current study are not publicly available since this was not requested at the Ethical Committee. Datasets are available from the corresponding author on reasonable request.

## Abbreviations

6MWD: 6 Min walk distance
6MWT: 6 Min walk test
APL: Absolute path length
ATS: American Thoracic Society
BAL: Balance training group
BMI: Body-Mass-Index
COPD: Chronic obstructive pulmonary disease
ERS: European Respiratory Society
FEV1: Forced expiratory volume in 1 s
GOLD: Global Initiative for Chronic Obstructive Lung Disease
LTOT: Long-term oxygen therapy
pO2: Partial oxygen pressure
PR: Pulmonary rehabilitation
STST: Sit-to stand test
WBV: Whole-body vibration
