# Supplementary material for: Whole-body vibration training versus conventional balance training in patients with severe COPD—a randomized, controlled trial
Source: Respir Res. 2021 May 4;22:138. doi: 10.1186/s12931-021-01688-x (PMC8097810; doi:10.1186/s12931-021-01688-x)
Supplement: Supplementary file 1 — Additional file 1. Additional tables and figures. [file 12931_2021_1688_MOESM1_ESM.docx]

**Additional File 1**

**Whole-body vibration training versus conventional balance training in patients with severe COPD – a randomized, controlled trial**

Authors: Rainer Gloeckl, Tessa Schneeberger, Daniela Leitl, Tobias Reinold,

Christoph Nell, Inga Jarosch, Klaus Kenn, Andreas R. Koczulla

**SUPPLEMENTAL METHODS**

Table S1: Description of the standardized pulmonary rehabilitation program

| **Rehabilitation content** | **description** |
| --- | --- |
| Optimization of medical treatment | Continuously adaptation of drugs, long-term oxygen therapy or non-invasive ventilation if necessary |
| Endurance training | Cycle endurance training was performed at 60% to 70% of peak work rate on 5 to 6 days per week for 10 to 20 minutes per session. |
| Strength training | Strength training was performed using resistance training machines. Following exercises were performed: leg press, knee extension, pull-down and push-down. If possible, following additional exercises were applied: butterfly forward/backward, rowing, back extension and abdominal trainer. Patients performed 3 sets per exercise at an individual intensity to reach momentary muscular failure after 15 to 20 repetitions. Resistance training usually took 30 to 45 minutes per session and was applied on 5 to 6 days per week. |
| Patient education | Patients visited 3 educational sessions per week for 60 minutes (on topics like disease management, device usage, oxygen therapy, physical activity, etc.) |
| Respiratory physiotherapy | Individually tailored chest physiotherapy using various techniques like breathing retraining, mucus clearance, cough techniques, energy conservation techniques and others was applied 2 to 4 times per week for 30 minutes each. |
| Activities of daily living training | Activities of daily living training (callisthenics) was applied 4 times per week for 30 minutes. |
| optional rehabilitation content | Following additional therapies were applied if necessary: occupational therapy, social services, nutritional counselling and psychological support. |

Table S2. Detailed description of WBV intervention according to description guidelines from Wuestefeld et al. PLoS ONE 2020; 15(7): e0235905

| ***Information about vibration*** |  |
| --- | --- |
| 1. The type of vibration (i.e., synchronous, side-alternating, other) | Side-alternating |
| 2. The units of the vibration parameters | Hertz |
| 3. The amplitude of the vibration (in mm) | 2 to 2,5 mm |
| 4. Which frequency (Hz) is used | 5, 18, 22 and 26 Hz as described in the main manuscript |
| 5. The position of each foot on the base of the vibration platform to calculate PDD or amplitude for side-alternating vibration | PDD: 4 to 5 mm |
|  |  |
| 6. Whether the frequency (Hz) is constant or variable | Constant during the exercise |
| 7. Whether manufacturer settings or own settings are used | Manufacturer settings were used |
| 8. How the vibration parameters are measured | - |
| 9. Whether the vibration is immediately full or increases slowly | Immediately full |
| 10. Which peak-to-peak displacement (PDD) of the vibration occurs | 4 to 5 mm |
| 11. Where on the platform the vibration parameters are measured | - |
| 12. Which peak-acceleration (in multiples of g) occurs | 27,1 g |
| 13. The definitions/explanations of the vibration parameters | - |
| 14. Vibration parameters should be measured with |  |
| • frequency and amplitude, *or* |  |
| • frequency, amplitude, and peak-to-peak displacement, *or* | See main manuscript |
| • the aid of 3D-accelerometers |  |
| a. with 3D-accelerometer at vibration platform and on participant, *and/or* | n.a. |
| b. 3D-accelerometer for vertical and horizontal acceleration | n.a. |
|  |  |
| ***Information about device*** |  |
| 15. Whether the device vibrates horizontal, vertical, side-alternating, waveform changing, or other) | Side-alternating |
| 16. Whether changes are made to the device (e.g., mounting a chair on it) | No |
| 17. The manufacturer, device specifications, and production type | Galileo Med L, type number: 8N057451 |
| 18. Whether a handrail is available | Yes |
|  |  |
| ***Information about administration*** |  |
| 19. Which posture or body position the participants take on during the vibration (e.g., sitting, standing, squatting) | See main manuscript for description |
| 20. Whether the position/posture changes during the WBV (static versus dynamic exercise) | See main manuscript for description |
| 21. The number of sessions where WBV was utilized | On average 7 to 8 sessions per subject |
| 22. The resting time between sessions of WBV | WBV was applied every second day |
| 23. The number of exposures to WBV within one session | 4 exercises with 2x1minutes duration (total: 8 minutes per session) |
| 24. Where the feet of the participants are placed | During bipodal exercises on markers 2 to 2,5 and during single-leg stance in the middle of the platform axis (marker 0) |
| 25. The total exposure time to WBV across all sessions | 56 to 64 minutes |
| 26. Where the hands of the participants are placed | In the beginning on the handrail then free standing |
| 27. The on vs. off-times of vibration (e.g., pauses and how long) within one session | 1 minute exercise alternating with 1 minute of rest (sitting) |
| 28. Which exact tools and aids were used during the WBV (e.g. type and size of dumbbells) | n.a. |
| 29. Whether the outcome measures (cognitive/physical) are assessed during or after the WBV | After (on a separate day) |
| 30. Whether an examiner was present to supervise the WBV administration | Each training session was individually supervised by a therapist |
| 31. Possible follow-ups to determine possible lasting effects | n.a. |
| 32. To report whether only parts of the subjects’ body are subjected to vibration (e.g., only the feet) | Subjects were standing on the platform |
| 33. The location of the intervention (e.g., hospital or gym) | Rehabilitation center |
| 34. To explain the decision which parts (e.g., only feet) of the participants are subjected to vibration and why | Patients with COPD have lower limb muscle dysfunction which impairs their walking ability. Therefore the main focus was to choose standing exercises. |
|  |  |
| ***Information about participants*** |  |
| 35. The participants’ height and weight | See baseline characteristics |
| 36. The participants’ footwear (shoes, socks, barefoot) during WBV with a detailed | Regular shoes |
| description |  |
| 37. Subjective experiences of participants before, during, or after the WBV |  |
| • Side effects/adverse effects | No |
| • Pain | No |
| • Dizziness | No |
| • (Dis)comfort | Some patients got dyspnoeic during exercise (due to COPD) |
| • Fatigue/exhaustion/tiredness | no |
| • Tingling/itching/burning sensations | Yes – a common sensation in almost all subjects |
| • Perceived exertion/effort (e.g., with Borg RPE scale) | n.a. |
| • Muscle soreness/weakness | Some patients felt muscle soreness the day after WBV training |
| • Headache | No |
| • Loss of balance | Yes (which was the aim of this study – to train balance) |
| 38. The participants’ fitness and activity levels | Impaired and low |
| 39. If, how, and for how long participants prepared for the WBV (e.g., stretching, muscle warm up) | No warm-up was performed |
| 40. Training history | unknown |


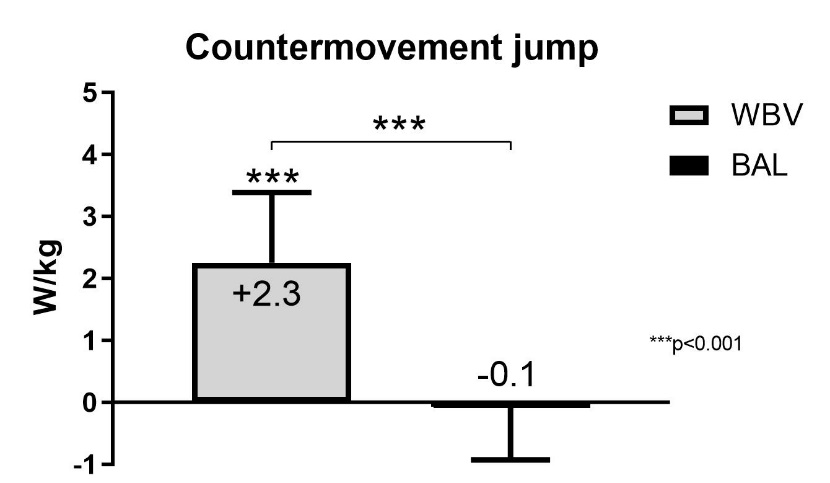


Figure S1. Changes in countermovement jump following a balance training using whole-body vibration (WBV) or conventional balance training (BAL) in patients with severe COPD an impaired functional status


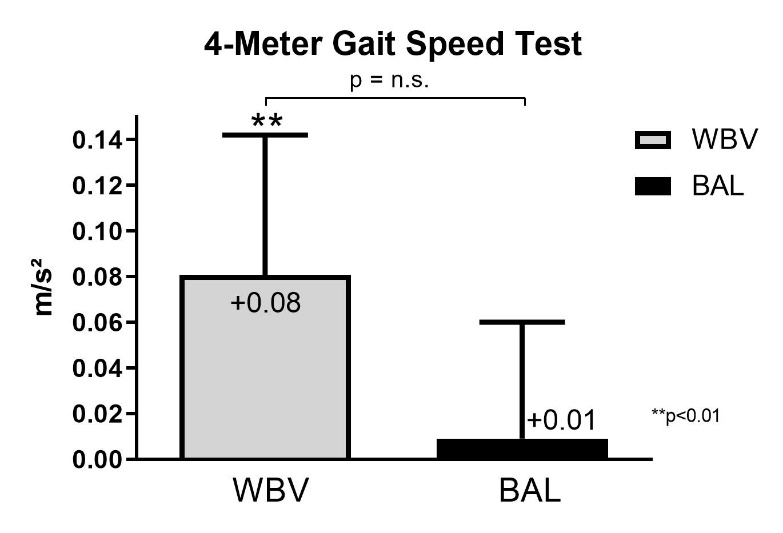


Figure S2. Changes in 4-m gait speed test jump following a balance training using whole-body vibration (WBV) or conventional balance training (BAL) in patients with severe COPD an impaired functional status


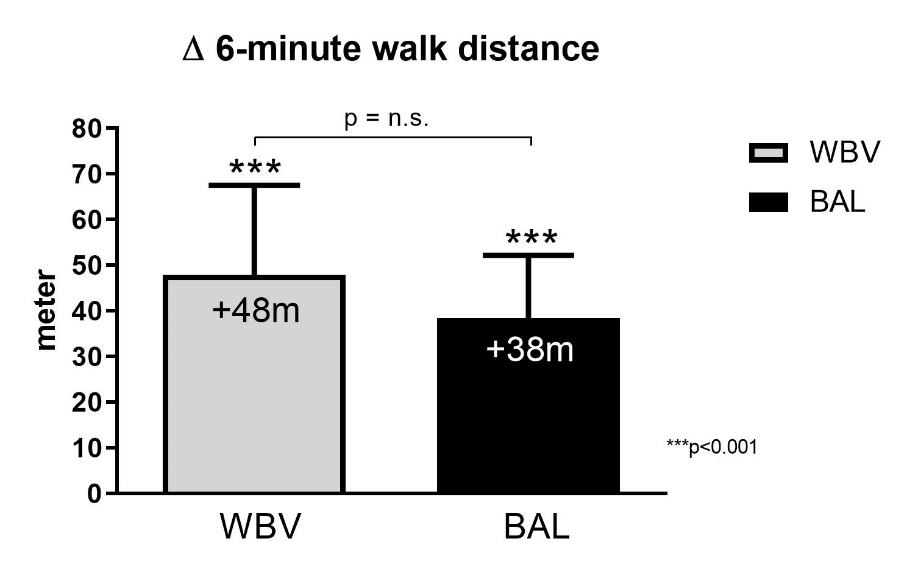


Figure S3. Changes in 6-minute walk distance following a balance training using whole-body vibration (WBV) or conventional balance training (BAL) in patients with severe COPD an impaired functional status


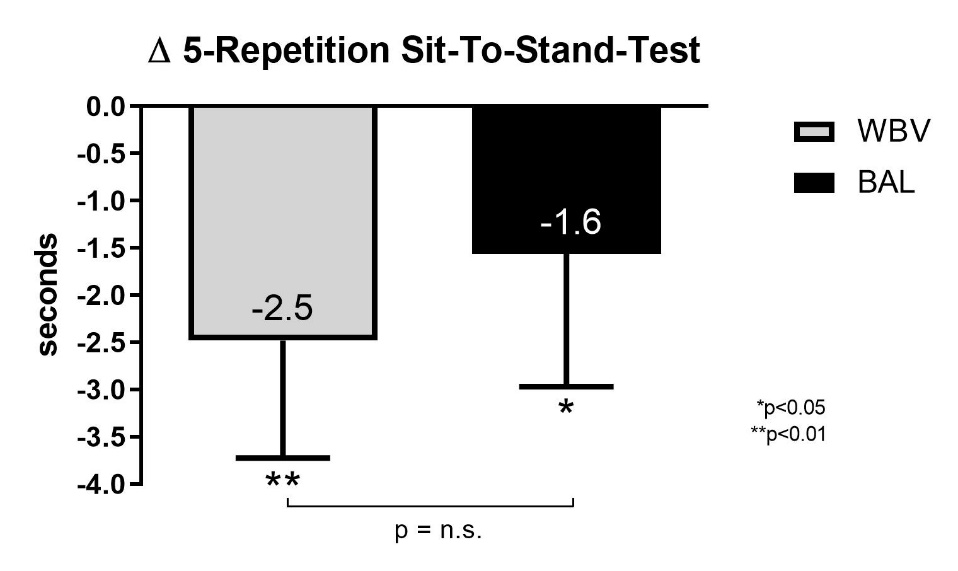


Figure S4. Changes in 5-repetition sit-to-stand test following a balance training using whole-body vibration (WBV) or conventional balance training (BAL) in patients with severe COPD an impaired functional status
